# Comparative efficacy and cognitive safety of magnetic seizure therapy and electroconvulsive therapy in major depressive disorder: a systematic review and meta-analysis

**DOI:** 10.3389/fpsyt.2026.1873016

**Published:** 2026-06-17

**Authors:** Kaipeng Fan, Xuekang Niu, Jun Zhao, Guohao Lin, Jiayang Qu, Qiaoqiao Wang, Lin Li

**Affiliations:** 1 Affiliated Mental Health Center & Hangzhou Seventh People’s Hospital, Zhejiang University School of Medicine, Hangzhou, Zhejiang, China; 2 Rehabilitation Assessment and Treatment Center, The Third Affiliated Hospital of Zhejiang Chinese Medical University, Hangzhou, Zhejiang, China

**Keywords:** cognitive safety, electroconvulsive therapy, magnetic seizure therapy, major depressive disorder, systematic review

## Abstract

**Background:**

Major depressive disorder (MDD) necessitates treatments that balance efficacy with tolerability. Electroconvulsive therapy (ECT) is highly effective but limited by cognitive side effects. Magnetic seizure therapy (MST), a more focal convulsive therapy, may offer a superior safety profile. This systematic review and meta-analysis directly compares the efficacy and cognitive safety of MST and ECT for MDD.

**Methods:**

We systematically searched PubMed, Embase, Cochrane CENTRAL, Web of Science, WanFang, and CNKI (inception to Nov 2025) for randomized and non-randomized controlled studies comparing MST and ECT in adults with MDD. Primary outcomes were antidepressant response and depression score changes. Secondary outcomes included cognitive function, reorientation time, and adverse events. Pooled effect estimates (RR, SMD, MD, OR) with 95% CIs were calculated.

**Results:**

13 studies (N = 607 participants) were included. MST and ECT demonstrated comparable clinical response (RR = 1.10, 95% CI: 0.94-1.27) and remission (RR = 1.04, 95% CI: 0.72-1.50) rates. Post-sensitivity analysis favored ECT for depression score change (SMD = 0.36, p=0.0001). MST was superior in preserving cognitive function (SMD = 1.19, p=0.005), enabling faster reorientation (MD=-16.72 min, p<0.00001), and reducing overall adverse event risk (OR = 0.23, p<0.00001), notably for memory loss, headache, and muscle pain. Seizure durations were shorter with MST.

**Conclusions:**

While MST and ECT had comparable response and remission rates, sensitivity analysis of depression score changes suggested potential superiority of ECT, warranting cautious interpretation. MST provided significantly better cognitive safety and tolerability, including fewer cognitive adverse events and faster reorientation. These findings support MST as a valuable alternative for MDD patients, especially when cognitive side effects are a primary concern.

This systematic review and meta-analysis was conducted and reported in strict accordance with the Preferred Reporting Items for Systematic Reviews and Meta-Analyses (PRISMA) 2020 guidelines (23).

**Systematic Review Registration:**

https://www.crd.york.ac.uk/PROSPERO/view/CRD420261277041, identifier CRD420261277041.

## 1 Introduction

Major depressive disorder (MDD) is a leading cause of global disability and a critical public health challenge (1, 2). Over one in five individuals is projected to experience at least one depressive episode (3). A cross-national study reported that the lifetime prevalence of MDD is 7.5% in males and 13.6% in females, with projected cumulative risks by age 75 of 20.1% and 34.0%, respectively (4). In addition, the COVID-19 pandemic significantly increased the global burden of MDD, particularly among women and young adults (5). Beyond its profound personal suffering, MDD inflicts a severe societal and economic toll (1, 6). Alarmingly, a vast treatment gap persists; an analysis of 15 countries revealed that while 41.8% of individuals with 12-month MDD accessed mental health services, only 23.2% of these received minimally adequate treatment, culminating in an overall 90% gap in effective treatment coverage (7). This chasm is driven by a combination of service underutilization and deficiencies in treatment quality or adherence, highlighting the urgent, unmet need for accessible, tolerable, and highly effective therapeutic strategies (8). In this context, neuromodulation therapies have assumed an increasingly prominent role in the management of MDD (9).

Electroconvulsive therapy (ECT) is widely regarded as an effective acute intervention for MDD, particularly in patients with psychotic features, high suicidality, or profound functional impairment (10, 11). Robust evidence from randomized controlled trials and meta-analyses has consistently demonstrated its superior antidepressant efficacy compared with pharmacotherapy and sham treatments (10–12). However, the clinical applicability of ECT is substantially limited by concerns regarding cognitive adverse effects, including acute disorientation as well as anterograde and retrograde memory impairment (13, 14), which remain a major barrier to treatment acceptance despite technical refinements (15). Magnetic seizure therapy (MST) has emerged as a novel convulsive neuromodulation technique aiming to preserve the antidepressant efficacy of ECT while reducing cognitive burden through more focal stimulation and limited involvement of medial temporal structures (9, 16). The first deliberate seizure induction using MST was demonstrated in non−human primates (17) and subsequently in patients with major depression (18). Early studies suggested that MST may offer comparable antidepressant outcomes with a more favorable cognitive safety profile (16, 19, 20). Subsequently, larger randomized controlled trials, including a double-blind trial by Deng et al. (21) and the recent non−inferiority CREST-MST trial (22) randomizing 239 patients, have provided higher−quality evidence generally supporting these findings. Nevertheless, existing evidence still shows heterogeneity in stimulation parameters, comparator ECT protocols, and outcome measures, and a comprehensive systematic synthesis of all available controlled studies remains warranted to quantify the overall effect sizes and to explore sources of variability.

The present systematic review and meta-analysis aims to compare the antidepressant efficacy and cognitive safety of magnetic seizure therapy versus electroconvulsive therapy in patients with major depressive disorder. By integrating evidence from randomized controlled trials and controlled clinical studies, this study seeks to provide a clearer evidence base to inform clinical decision-making, guide future research priorities, and ultimately contribute to optimizing treatment pathways for patients with MDD.

## 2 Methods

This systematic review and meta-analysis was conducted and reported in strict accordance with the Preferred Reporting Items for Systematic Reviews and Meta-Analyses (PRISMA) 2020 guidelines (23). The study protocol was prospectively registered on the International Prospective Register of Systematic Reviews (PROSPERO) under the registration number CRD420261277041.

### 2.1 Search strategy and information sources

A comprehensive and systematic literature search was performed to identify all relevant studies comparing Magnetic Seizure Therapy (MST) and Electroconvulsive Therapy (ECT) for Major Depressive Disorder (MDD). With the assistance of a senior research librarian, we developed search strategies tailored to each database’s syntax. In order to avoid missing any relevant literature as much as possible, the search strategy combined controlled vocabulary terms (e.g., MeSH in PubMed) and free-text keywords related to two core concepts: Magnetic Seizure Therapy (e.g., “Magnetic Seizure Therapy”, “MST”) and Major Depressive Disorder (e.g., “Depressive Disorder, Major”, “Depression”, “Treatment Resistant Depression”). We searched the following six electronic databases from their inception until November 4, 2025: PubMed/MEDLINE, Embase, Cochrane Central Register of Controlled Trials (CENTRAL), Web of Science Core Collection, WanFang and CNKI. No language or publication status restrictions were applied. The complete search history for each database was provided in **Supplementary File 1**. To ensure literature saturation, we also manually screened the reference lists of all included studies and relevant systematic reviews.

### 2.2 Eligibility criteria

Studies were selected based on the following predefined criteria: Population: Adult patients diagnosed with major depressive disorder according to standardized diagnostic criteria (e.g., DSM or ICD); Intervention: Magnetic seizure therapy; Comparator: Electroconvulsive therapy; Outcomes: At least one predefined outcome related to antidepressant efficacy [e.g., Hamilton Depression Rating Scale (HAMD), Montgomery-Asberg Depression Rating Scale (MADRS)] or cognitive safety [e.g., Montreal Cognitive Assessment (MoCA), Repeatable Battery for the Assessment of Neuropsychological Status (RBANS) scores]; Study Design: Randomized controlled trials (RCTs), non-randomized controlled trials, and prospective or retrospective comparative cohort studies. Case reports, case series, reviews, conference abstracts without full data, and non-comparative studies were excluded.

### 2.3 Study selection and data extraction

All records identified through database searching were imported into Endnote (24) for deduplication and management. The selection process was conducted independently by two authors. First, titles and abstracts were screened against the eligibility criteria. Second, the full texts of potentially relevant articles were retrieved and assessed in detail. Any discrepancies at either stage were resolved through discussion or by consulting a third senior author. The reasons for excluding studies at the full-text stage were recorded. Extracted data included study characteristics (author, year, country, study design), participant demographics, treatment parameters, outcome measures, and follow-up duration. Detailed characteristics of the included studies are summarized in the study characteristics table.

### 2.4 Risk of bias assessment

The methodological quality of included randomized controlled trials was independently assessed by two authors using the Cochrane Risk of Bias 2 (ROB 2) tool (25), which evaluates bias across five domains: randomization process, deviations from intended interventions, missing outcome data, outcome measurement, and selection of the reported result. Overall risk of bias judgments were categorized as low risk, some concerns, or high risk. Any disagreements in assessment were resolved by consensus.

### 2.5 Statistical analysis

Meta-analyses were performed using Review Manager (RevMan) 5.4 (26) For continuous outcomes, when the measurement units of the outcome were entirely consistent and the same assessment tool was used across studies in a meta-analysis, the weighted mean difference (WMD) was preferentially selected. Conversely, when the measurement units differ or different assessment tools were used, the standardized mean difference (SMD) was the preferred choice. For the analysis of dichotomous variables, the risk ratio (RR) is used for comparing efficacy rates (e.g., response rates), whereas the odds ratio (OR) is typically chosen for the analysis of adverse events. All results obtained were reported with 95% confidence intervals (CI). For the statistical model, a fixed-effect model is employed when heterogeneity is low, while a random-effects model is adopted when heterogeneity is substantial. Heterogeneity among studies was determined by Q test and I^2^ statistics [Cochrane book 9.5.2 Identifying and measuring heterogeneity, 0%–40%: might not be important; 30%–60%: may represent moderate heterogeneity*; 50%–90%: may represent substantial heterogeneity*; 75%–100%: considerable heterogeneity*] (27). Sensitivity analyses were conducted by sequentially excluding individual studies to examine the robustness of the primary outcome. Publication bias was evaluated visually using funnel plots for the primary outcome.

## 3 Results

### 3.1 Study selection

**Figure 1:**
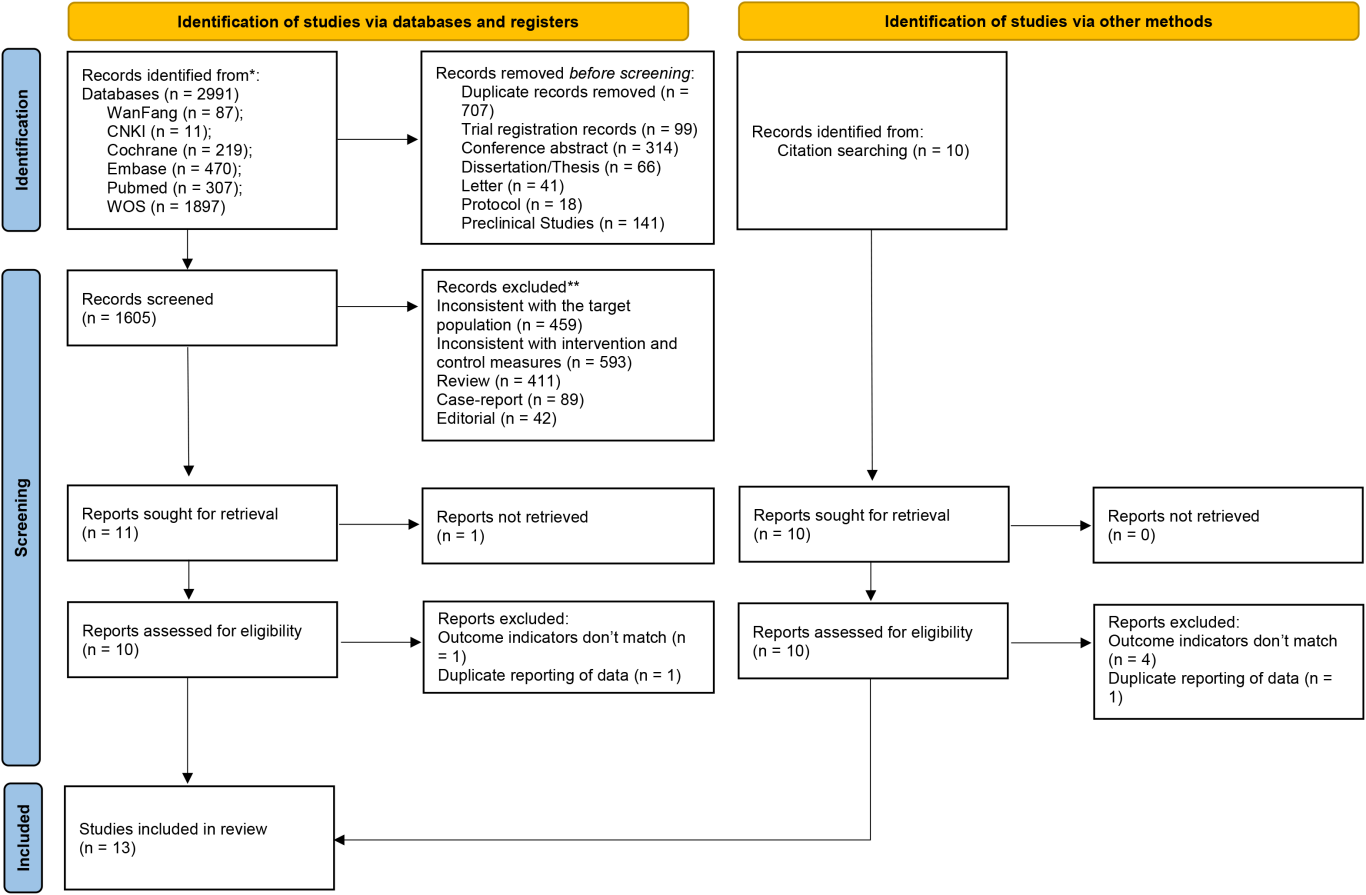
Literature selection and inclusion process.

The PRISMA flow diagram (**Figure 1**) summarizes the study selection process. A systematic search of six electronic databases (WanFang, CNKI, Cochrane CENTRAL, Embase, PubMed, and Web of Science) yielded 2,991 records. An additional 10 records were identified through citation searching of included studies and relevant reviews. After removing duplicate records and ineligible publication types during the initial screening, a total of 1,605 records were screened by title and abstract. Of these, 1,594 records were excluded because they did not meet the eligibility criteria. The remaining 21 reports were sought for full-text retrieval, of which 1 could not be obtained. The full texts of the remaining 20 reports were assessed for eligibility. Among these, 5 were excluded due to mismatched outcome indicators, and 2 were excluded because it represented duplicate reporting of data from an already included study, as detailed in **Supplementary file 2**. Consequently, a total of 13 studies met all predefined eligibility criteria and were included in the meta-analysis.

### 3.2 Characteristics of included studies

The meta-analysis included 13 studies (8 randomized controlled trials [RCTs] and 5 non-randomized studies) published between 2006 and 2025, involving a total of 607 participants. Among them, 297 patients were treated with MST and 310 with ECT. All studies enrolled adult patients diagnosed with a major depressive episode, most with treatment-resistant depression (TRD). Concomitant antidepressant use was common. The stimulation parameters for MST were highly consistent across studies, predominantly utilizing 100 Hz frequency and maximal output intensity targeted at the vertex. In contrast, ECT protocols were heterogeneous, employing varied electrode placements (right unilateral, bifrontal, bitemporal) and dosing methods. All studies assessed depression severity using the Hamilton Depression Rating Scale (HAMD), and most evaluated cognitive function. Detailed study characteristics are available in **Table 1**.

**Table 1:** Characteristics of included studies.

Study ID	Country	Study Design	Population	Diagnostic Criteria	Antidepressants	MST Group									ECT Group							Outcomes & Tools	Assessment Timepoints
						N	Age (years)	Duration of illness	Coil Type	Target	Frequency (Hz)	Intensity	Pulses/Duration	Treatment Course	N	Age (years)	Duration of illness	Charge (mC)	Pulse Width	Electrode Placement	Treatment Course		
Wang 2025b (28)	China	Evaluator-blinded RCT	Adolescents (13–18 yrs) with TR-MDD or high suicide risk	DSM-V	Yes (continued SSRI)	60 (randomized), 45(completed)	15.20 ± 1.75	14.52 ± 13.06 (month)	/	Cz (10–20 system)	100 Hz	100% intensity	8–10 s/session	3 times/week for 12–16 sessions	60 (randomized), 50 (completed)	15.15 ± 1.64	12.42 ± 13.28 (month)	Age-based percentage method	/	Bilateral	3 times/week for 12–16 sessions	BDI-II; MoCA; C-SSRS; CTCAE v5.0; Reorientation time	Baseline, Day 7 post-final treatment.
Yang 2025 (29)	China	RCT	Inpatients with MDE (MDD or bipolar disorder)	DSM-V	Yes (SSRIs)	20	29 (21, 46)	78 (36, 192) (month)	/	/	100 Hz	100% output	1st: 4s, +2s each session, max 10s	3 times/week for 4 weeks (12 sessions)	20	31 (21, 55)	48 (7, 102) (month)	Determined by age (0.8 × age × 100%)	1.0 ms	/	3 times/week for 4 weeks (12 sessions)	HAMD-17; RBANS (total & factor scores)	Pre- and post-treatment.
Deng 2024 (21)	USA	Double-blind RCT	Aged 18-90, referred for ECT, MDE in MDD/bipolar, baseline HDRS-24≥18.	DSM-IV-TR (SCID interview)	No (tapered/washed out pre-treatment)	35 (randomized)	47.7 ± 15.6	135.2 ± 208.3 (weeks)	Double-cone circular coil	Vertex	100 Hz	100% max output	10 s (titrated from 5s)	3 times/week until remission criteria, plateau, or <25% improvement after 8 sessions. Avg: 9.0 sessions.	38 (randomized)	48.2 ± 12.8	114.5 ± 129.4 (weeks)	6 × seizure threshold	Ultrabrief pulse	RUL	3 times/week until remission criteria, plateau, or <25% improvement after 8 sessions. Avg: 6.7 sessions.	HDRS-24; IDS; CGI-I; GAF; MMSE; Columbia ECT Side Effects; Reorientation time; AMT	Baseline, each treatment morning, 24–72 hrs post-final, follow-up (first 2 months: 2/month, then 1/month, up to 6 months).
EI-Deeb 2020 (30)	Egypt	Open-label RCT	MDD patients, aged 18-65, with indication for convulsive therapy (e.g., suicidality, psychosis).	DSM-IV-TR	No (drug-free ≥6 wks or naïve)	30	39.07 ± 12.85	7.73 ± 5.66 (month)	Circular coil	Vertex	100 Hz	100% max output	10 s	5 sessions, 2 times/week	30 (15 RUL, 15 BT)	BT:38.80 ± 14.0 RUL:39.60 ± 12.32	BT:6.00 ± 5.41 RUL:5.73 ± 4.03 (month)	RUL: age%; BT: (age/2)%	Brief (0.5 ms)	RUL (15) & BT (15)	5 sessions, 2 times/week	HAMD-21; BDI; TRO; Wechsler Memory Scale; Wisconsin Card Sorting Test; Columbia ECT Side Effects Schedule	Baseline, after sessions 1, 3, 5.
Fitzgerald 2018 (20)	Australia	Double-blind RCT	Patients with TRD (Stage II), moderate-severe depression	DSM-IV	Yes, continued use allowed	18 (final analysis)	44.6 ± 14.8	22.7 ± 14.3 (years)	Bilateral double-cone coil	Vertex	100 Hz	100% machine output	Titration: +2s, Treatment: threshold+4s, max 10s	Up to 15 sessions, 3 times/week	19	47.2 ± 16.1	27.6 ± 14.4 (years)	3 × threshold	/	RUL	Up to 15 sessions, 3 times/week	HAMD; IDS-C; QIDS; Cognitive tests (AMI, Digit Symbol, Stroop, etc.)	Baseline, after sessions 6, 9, 12, 15, end of treatment.
Polster 2015 (31)	Germany	Open-label RCT	Patients with TRD	DSM-IV (MDE)	Yes (stable 1 month before & during)	10	43.7 ± 11	4.1 ± 4 (years)	Twin coil (13 cm wide)	Vertex	100 Hz	~4 T (coil surface)	Avg 5–8 s	10–12 sessions, 2 times/week	10	54.7 ± 13	3.1 ± 3 (years)	5 × seizure threshold	Biphasic, square-wave, 0.5 ms	RUL	10–12 sessions, 2 times/week	Acute memory tests (Delayed & Cued recall)	Treatment vs. control days: morning (learning) & noon (recall).
Kayser 2017 (32)	Germany	Open-label RCT	Patients with TRD (MDD, BPI, BPII)	DSM-IV-TR	Yes (stable ≥4 wks)	10 (completed)	45 ± 14	/	Twin coil	Cz (10–20)	100 Hz	100% max output	Max 800 pulses, 8 s	8–12 sessions, 2 times/week	10 (completed)	55 ± 12	/	RUL: 6×threshold; BL: 3×threshold	/	RUL (9), Bifrontotemporal (1)	8–12 sessions, 2 times/week	HRSD-28; Seizure characteristics (postictal suppression)	Within 7 days pre- and post-treatment.
Kayser 2011 (19)	Germany	Open-label RCT	Patients with TRD	DSM-IV	Yes (as add-on)	10	50.0 ± 11.9	12.7 ± 3.6 (month)	Twin coil	Vertex	100 Hz	100% output	Max 600 pulses, 6 s	12 sessions, 2 times/week	10	49.0 ± 11.0	13.3 ± 4.1 (month)	3 × seizure threshold	Biphasic square-wave	RUL	12 sessions, 2 times/week	MADRS; HDRS-28; HAMA; BDI; SCL-90; Neuropsychological tests; Recovery/Orientation time	Baseline, post-treatment (1 month ±3 days after final), multiple cognitive assessments during treatment.
Wang 2025a (33)	China	Controlled	Inpatients with TRD, aged 18–65	ICD-11 MDD + TRD criteria	Yes	42	28.6 ± 13.8	15.9 ± 13.2 (month)	Double-cone coil	Vertex (10–20 system)	100 Hz	100% output	8–10 s	3 times/week for 12 sessions (up to 16)	44	30.7 ± 14.7	18.2 ± 13.4 (month)	Initial: (age × 0.8)%; Max: (2 × age)%	/	Bitemporal	3 times/week for 12 sessions (up to 16)	HAMD; MoCA; Reorientation time; Length of stay; Columbia ECT Side Effects Questionnaire	Baseline (within 1 wk pre-treatment); Post-treatment (48–72 hrs after final session).
Zhang 2020 (34)	China	Evaluator-blinded, non-randomized	Depressive episode patients, aged 18–60	DSM-IV-TR	Yes (SSRIs allowed, stable dose)	18	29.00 ± 8.32	3.77 ± 3.77 (years)	Circular coil	Vertex	100 Hz	100% max output	Max 10 s	Sessions on days 1, 2, 3, 5, 7, 9 (6 sessions total)	27	32.78 ± 8.84	4.47 ± 5.42 (years)	Determined by half-age method	/	Bifrontal	Sessions on days 1, 2, 3, 5, 7, 9 (6 sessions total)	HAMD-17; HAMA; RBANS; Response/Remission rates; Seizure duration; Recovery times (breathing, consciousness, orientation)	Baseline, after 3rd session (Day 3), after 6th session (Day 9).
White 2006 (35)	USA	Open-label case-control	Adult patients with severe depression	/	/	10	48 ± 4	/	Custom-modified Magstim device (16 booster modules)	/	50 Hz	2 T (coil surface)	8 s	10–12 sessions over 3–4 weeks	10	49 ± 6	/	2.5 × seizure threshold	0.5 ms	Bitemporal	10–12 sessions over 3–4 weeks	Ham-D; BIS; Reorientation time; Cardiovascular response; Seizure duration (EEG & motor)	Baseline, post-treatment.
Atluri 2018 (36)	Canada	Prospective observational	75 TRD patients; 55 healthy controls.	DSM-IV MDD	Yes, most on antidepressants or benzodiazepines.	24	46.8 ± 15.8	19.0 ± 12.0 (years)	Twin Coil (MagVenture)	Bifrontal (F3, F4; DMPFC)	100 Hz (12), 60 Hz (1), 50 Hz (2), 25 Hz (9)	Max E-field intensity	/	Up to 24 sessions or until remission, 2–3 times/week.	22	42.0 ± 13.4	20.3 ± 13.7 (years)	/	RUL ultrabrief pulse (RUL-UB) or Bitemporal brief pulse (BL)	RUL or Bitemporal	2–3 times/week until remission/plateau.	HRSD-17; MoCA; BDI; MADRS; SSI	Within 1 week pre-treatment; within 2 weeks post-treatment.
Soehle 2014 (37)	Germany	Prospective observational	Patients with TRD	DSM-IV-TR	Yes	10	45 ± 14	/	Twin coil	/	100 Hz	100% amplitude	Max 8 s	~10–12 sessions, 2 times/week	10	55 ± 12	/	/	/	Right unilateral (9), Bilateral (1)	~10–12 sessions, 2 times/week	BIS monitoring; Recovery times (breathing, eye-opening); Seizure duration (EEG & motor)	Baseline, pre-treatment, within 10 min post-treatment.

AMI, Autobiographical Memory Interview; BDI, Beck Depression Inventory; BIS, Bispectral Index; BL, Bitemporal; BT, Bitemporal; CGI-I, Clinical Global Impression-Improvement; CFQ, Cognitive Failures Questionnaire; C-SSRS, Columbia-Suicide Severity Rating Scale; CTCAE, Common Terminology Criteria for Adverse Events; DMPFC, Dorsomedial Prefrontal Cortex; ECT, Electroconvulsive Therapy; GAF, Global Assessment of Functioning; Ham-D/HAMD, Hamilton Depression Rating Scale; HDRS/HRSD, Hamilton Depression Rating Scale (item number specified); IDS, Inventory of Depressive Symptomatology; MADRS, Montgomery-Åsberg Depression Rating Scale; MDE, Major Depressive Episode; MDD, Major Depressive Disorder; MMSE, Mini-Mental State Examination; MoCA, Montreal Cognitive Assessment; MST, Magnetic Seizure Therapy; QIDS, Quick Inventory of Depressive Symptomatology; RCT, Randomized Controlled Trial; RBANS, Repeatable Battery for the Assessment of Neuropsychological Status; RUL, Right Unilateral; SSI, Scale for Suicidal Ideation; AMT, Autobiographical Memory Test; TRD, Treatment-Resistant Depression; TRO, Test of Reorientation.

### 3.3 Risk of bias assessment

**Figure 2:**
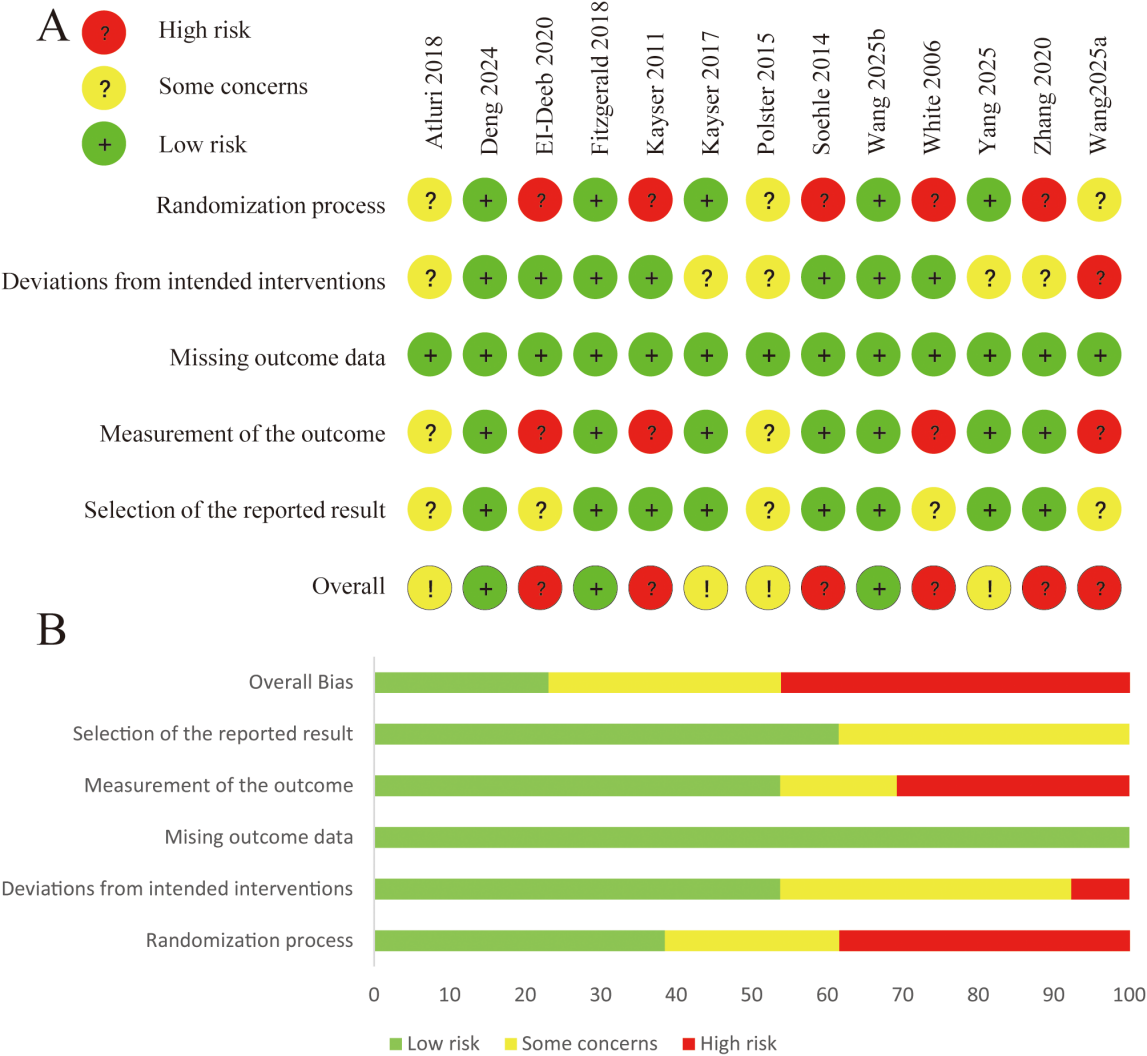
Risk of bias graph. **(A)** each risk of bias item for each included study. **(B)** each risk of bias item presented as percentages across all included studies.

A risk of bias assessment was performed on the 13 included studies (**Figure 2**). 3 studies were judged as “low risk” (20, 21, 28), 4 as “some concerns” (29, 31, 32, 36), and 6 as “high risk” (19, 30, 33–35, 37). Elevated risk in the “randomization process” domain was noted, attributable to the inclusion of 5 non-randomized studies (33–37) and 3 with unclear randomization (19, 30, 31). In addition, blinding of treating personnel was not feasible due to procedural differences. Participant blinding is achievable under anesthesia, but several included studies did not implement it adequately, introducing potential performance and detection bias. In the “measurement of the outcome” domain, four studies (28.6%) were at high risk and two (14.3%) raised some concerns, primarily because key outcome measures involved subjective assessment. Detailed assessments for each domain and study are provided in **Supplementary File 3**.

### 3.4 Primary outcome

**Figure 3:**
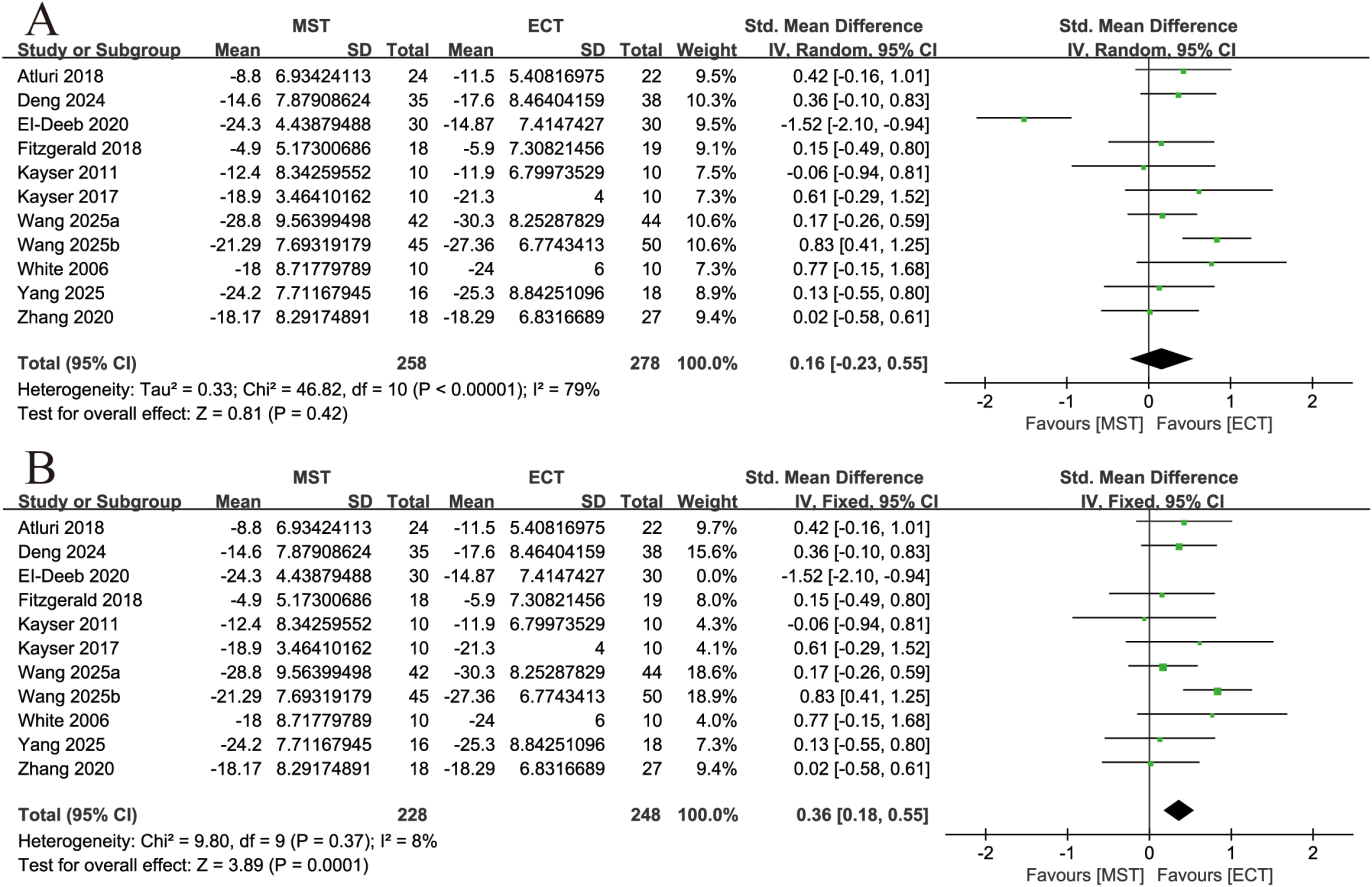
Forest plot of primary indicators. **(A)** Pooled results of the change in depression scale scores prior to sensitivity analysis. **(B)** Pooled results of the change in depression scale scores after sensitivity analysis.

Meta-analysis of the change in depression scale scores across 11 studies showed no statistically significant difference between MST and ECT prior to sensitivity analysis (SMD: 0.16, 95% CI: -0.23 to 0.55, p = 0.42), though with high heterogeneity (I² = 79%) (**Figure 3A**). Subsequent sensitivity analysis identified one study (30) that reported an effect size strongly favoring MST (SMD = -1.52) and was an outlier within the overall dataset. The removal of this study significantly altered the pooled estimate. The analysis of the remaining 10 studies yielded a statistically significant result, with heterogeneity further reduced (I² = 8%), indicating superior antidepressant efficacy for ECT (SMD: 0.36, 95% CI: 0.18 to 0.55, p = 0.0001) (**Figure 3B**).

**Figure 4:**
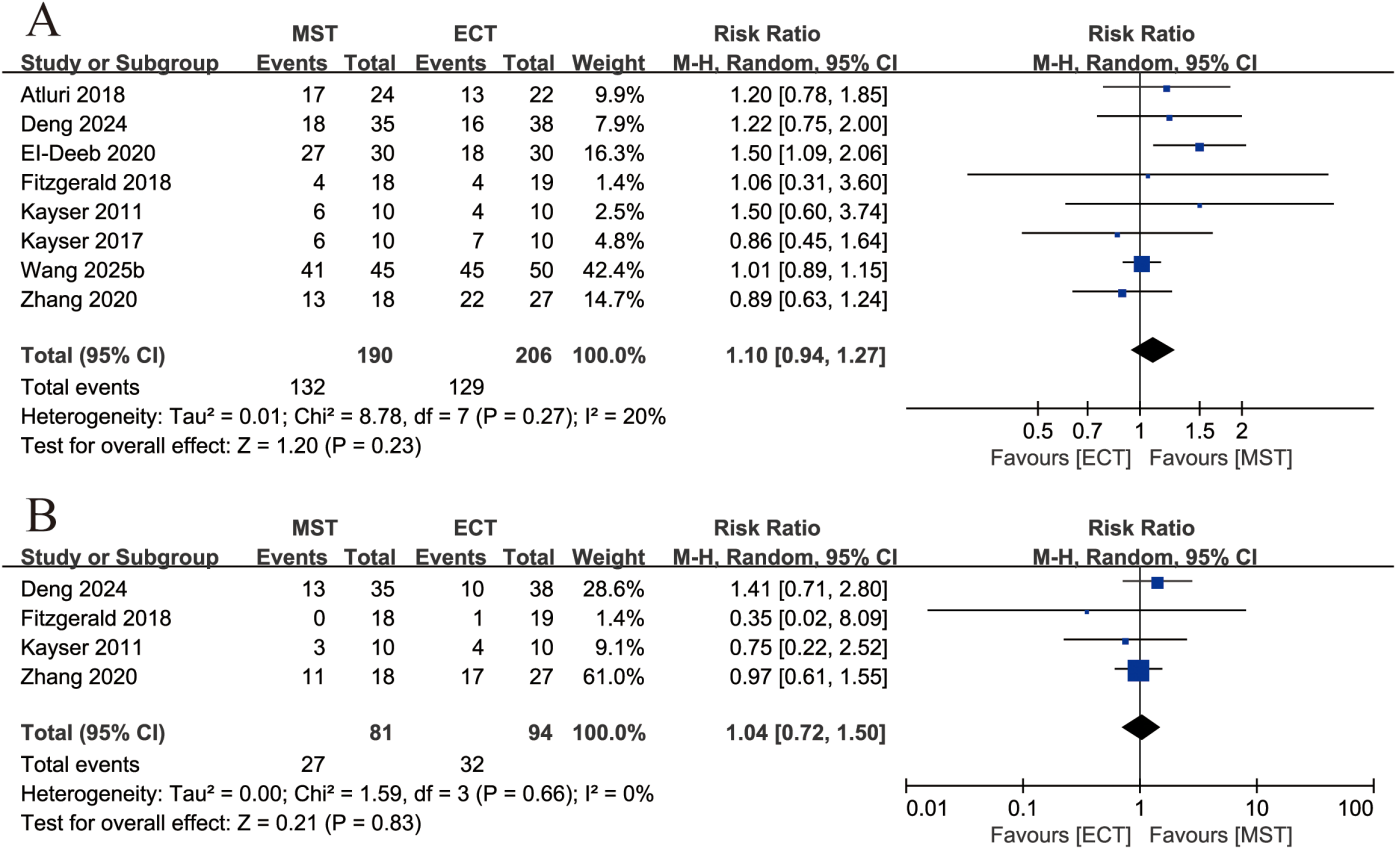
Forest plot of treatment response and remission rates. **(A)** Response number; **(B)** Remission number.

A total of 8 studies involving 396 participants (MST: n=190; ECT: n=206) reported data on treatment response rates. Meta-analysis showed no statistically significant difference between the MST and ECT groups. The pooled risk ratio (RR) was 1.10 (95% CI: 0.94 to 1.27, p = 0.23), indicating comparable rates of clinical response. Heterogeneity among studies was low (I² = 20%) (**Figure 4A**). Pooled analysis of 4 studies (total n=175; MST: n=81, ECT: n=94) also revealed no significant difference in achieving remission between the two interventions. The pooled RR was 1.04 (95% CI: 0.72 to 1.50, p = 0.83). Heterogeneity was negligible (I² = 0%) (**Figure 4B**).

### 3.5 Secondary outcomes

**Figure 5:**
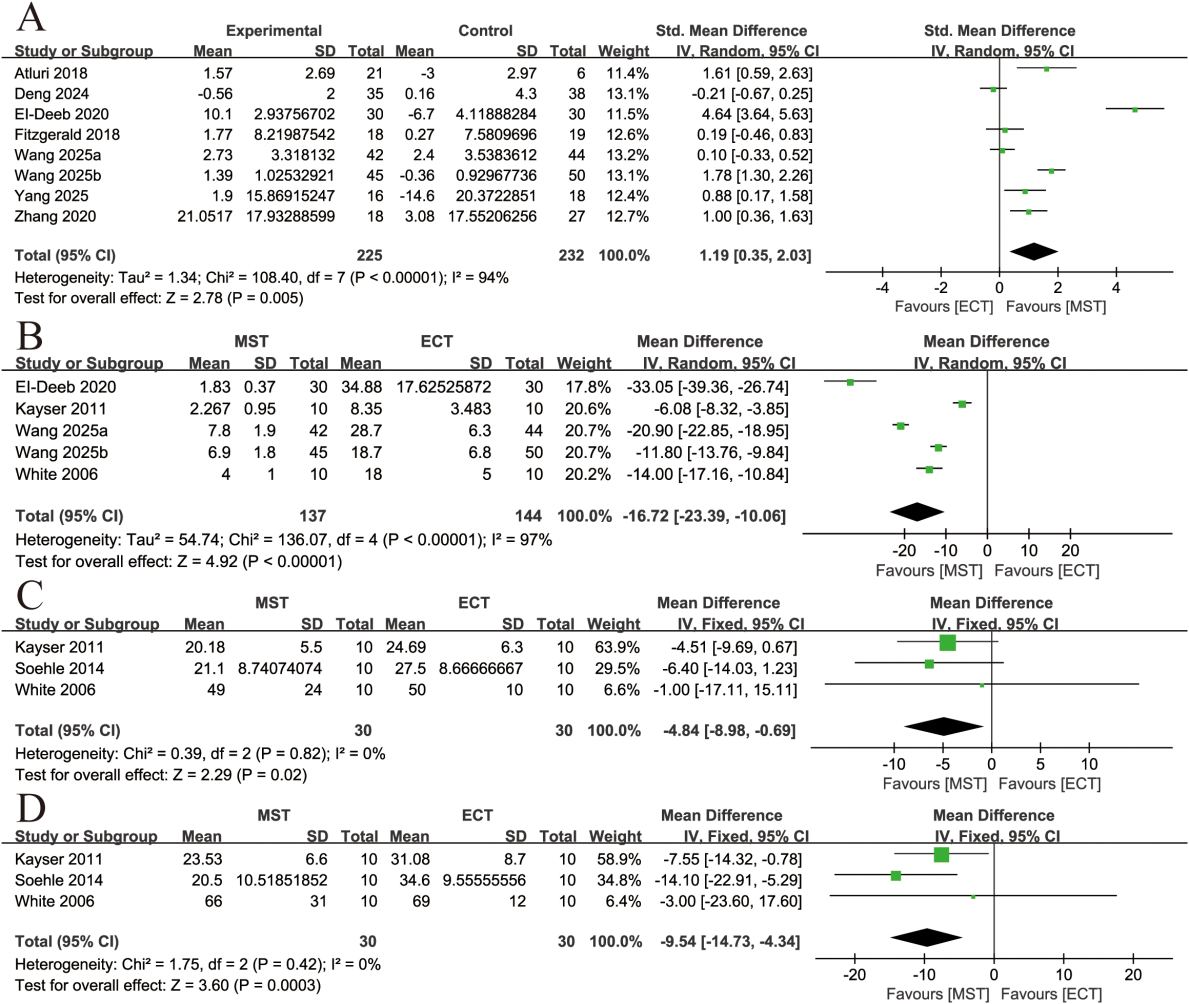
Forest plot of secondary indicators. **(A)** Cognitive Ability Scores; **(B)** Reorientation time; **(C)** Motor Seizure Duration; **(D)** EEG Seizure Duration.

The impact of MST and ECT on cognitive function was evaluated by pooling the change in cognitive ability scores from 8 studies, involving a total of 457 participants (225 in the MST group and 232 in the ECT group). The meta-analysis demonstrated a statistically significant advantage for MST. The pooled SMD was 1.19 (95% CI: 0.35 to 2.03; p = 0.005), indicating that patients treated with MST experienced significantly greater improvement or less decline in cognitive scores compared to those treated with ECT (**Figure 5A**). It is important to note that given the limited number of studies reporting data for individual cognitive domains (e.g., memory, executive function, attention), we performed an exploratory pooled analysis across all available cognitive measures using standardized mean differences (SMD) and a random−effects model. This approach allows the combination of different instruments under the assumption that they reflect a common latent construct of global cognitive change. However, the results should be interpreted with caution, as reflected by the considerable statistical heterogeneity (I² = 94%).

Analysis of reorientation time across 5 studies (n=281) showed that patients receiving MST regained orientation significantly faster than those receiving ECT. The pooled mean difference was -16.72 minutes (95% CI: -23.39 to -10.06, p < 0.00001), indicating a clear advantage for MST. Similar to the cognitive score outcome, this result exhibited very high heterogeneity (I² = 97%), suggesting considerable variation between studies, and the point estimate should be interpreted cautiously (**Figure 5B**).

Pooled data from 3 studies (n=60) revealed that motor seizures were significantly shorter in the MST group compared to the ECT group, with a mean difference of -4.84 seconds (95% CI: -8.98 to -0.69, p = 0.02). Heterogeneity was low (I² = 0%) (**Figure 5C**). Similarly, EEG seizure duration was shorter in the MST group. The analysis of 3 studies (n=60) yielded a mean difference of -9.54 seconds (95% CI: -14.73 to -4.34, p = 0.0003), with low heterogeneity (I² = 0%) (**Figure 5D**).

### 3.6 Adverse events

**Figure 6:**
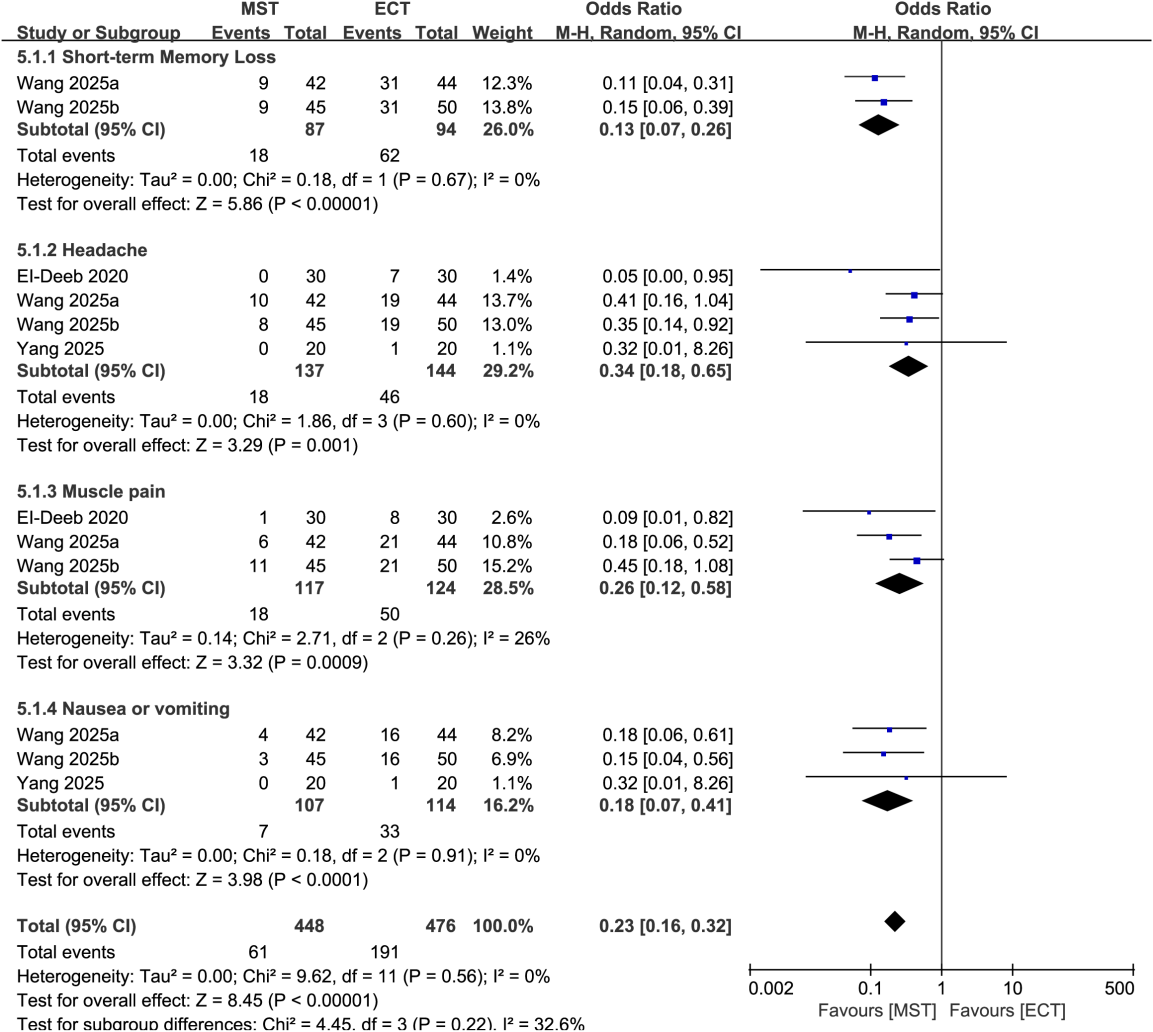
Forest plot of adverse events.

The meta-analysis demonstrated a significantly more favorable safety profile for MST compared to ECT (**Figure 6**). The quantitative synthesis, based on data from 4 studies, showed that the risk of experiencing any adverse event was substantially lower in the MST group (OR = 0.23, 95% CI: 0.16 to 0.32, p < 0.00001), with no significant heterogeneity (I² = 0%). This advantage was consistent across specific adverse events: short-term memory loss (OR = 0.13, 95% CI: 0.07 to 0.26), headache (OR = 0.34, 95% CI: 0.18 to 0.65), muscle pain (OR = 0.26, 95% CI: 0.12 to 0.58), and nausea or vomiting (OR = 0.18, 95% CI: 0.07 to 0.41). Descriptive analyses from other studies further supported these quantitative findings, reporting a higher incidence of subjective complaints (e.g., headache, confusion) and serious adverse events (e.g., hospitalization for worsened depression) in ECT groups, alongside qualitative statements highlighting fewer cognitive side effects with MST **Supplementary File 4**. These integrated results indicate that MST offers a superior tolerability profile with a markedly lower burden of common and cognitive side effects.

### 3.7 Publication bias

Funnel plot analysis for depression score change revealed asymmetry in the initial analysis including all 11 studies, with one outlying study (30) reporting a strongly negative SMD (−1.52) that deviated notably from the pooled effect (**Supplementary File 5A**). After excluding this study in a sensitivity analysis, the funnel plot of the remaining 10 studies displayed a roughly symmetrical inverted−funnel shape (**Supplementary File 5B**), indicating no significant publication bias among the remaining 10 studies.

## 4 Discussion

This systematic review and meta-analysis integrated evidence from 13 studies comparing MST and ECT for the treatment of MDD. The main findings are as follows: Regarding antidepressant efficacy, the primary analysis demonstrated no statistically significant difference between the two interventions. However, sensitivity analysis suggested that after the exclusion of one outlier study (30), ECT might demonstrate statistically superior short-term efficacy, although the clinical significance of this difference requires cautious interpretation. Concerning the core safety outcomes, MST exhibited clear and significant advantages: it was associated with better overall cognitive preservation (despite considerable heterogeneity), significantly faster reorientation time, and a markedly lower risk of both total and specific adverse events—most notably short-term memory impairment, headache, and muscle pain. MST induced significantly shorter motor and EEG seizure durations than ECT, while maintaining comparable clinical efficacy. No differences were observed between the groups in terms of response or remission rates.

From a rehabilitation perspective, preservation of cognitive function is not a secondary consideration but a core determinant of functional recovery, social reintegration, and quality of life (38, 39). The observed advantages of MST in cognitive ability scores, reorientation time, and adverse event profiles suggest that MST may better align with recovery-oriented treatment goals, especially in working-age adults and older patients who are vulnerable to cognitive decline. Thus, MST may serve not merely as a technical refinement of seizure therapy, but as a clinically meaningful alternative for a subset of patients with major depressive disorder.

The central finding of comparable clinical response rates, despite the observed shorter seizure duration in MST, invites a critical mechanistic inquiry. It has long been suggested that short seizures may be less clinically effective than long seizures (40). A large cohort study in ECT has indeed shown that the lowest remission rate, 27.2%, was observed in those with seizures lasting less than 20 seconds, whereas the highest remission rate was observed in those with seizures lasting 60 to 69 seconds (41). Our analysis confirms that MST seizures are consistently shorter in both motor and EEG manifestations than those induced by ECT. One possible explanation is a dosage mismatch: most MST protocols used fixed 100 Hz stimulation at maximal stimulator output, whereas ECT was typically dosed at multiples (e.g., 6 ×) above seizure threshold. Recent evidence suggests that 100 Hz may be inefficient for seizure induction with MST, and lower frequencies (e.g., 25–50 Hz) may yield lower seizure thresholds, thereby allowing a higher relative dose and potentially enhancing efficacy (42). Thus, the comparative efficacy of MST might be underestimated. Future studies should systematically explore the parameter space of MST to maximize its therapeutic index.

Moreover, accumulating evidence suggests that the relationship between seizure duration and clinical efficacy may differ fundamentally between MST and ECT due to their distinct biophysical mechanisms. Neurophysiological and modeling studies indicate that MST induces seizures that are more spatially focal and predominantly confined to superficial cortical regions, with reduced propagation to medial temporal structures such as the hippocampus, which are closely linked to memory formation (14, 16, 43, 44). This spatial focality stems not simply from bypassing scalp impedance, but from fundamental biophysical differences: MST induces tangential electric fields that drop off rapidly with distance from the coil, resulting in negligible field strength in deep brain structures, whereas ECT induces radial fields that are strong throughout the entire brain (43, 45–47). These characteristics likely underlie the shorter, more self−limited seizure activity observed with MST. In contrast, ECT delivers diffuse electrical stimulation that readily engages deeper limbic structures, including the hippocampus and amygdala, as demonstrated by electric field modeling (43, 47) and direct intracranial recordings in non−human primates (44), resulting in longer and more generalized seizures. Such seizure patterns have been consistently associated with greater cognitive disruption, particularly in memory domains (19). Therefore, the shorter seizure duration with MST may be an inherent feature of its focal mechanism rather than an indicator of subtherapeutic dosing.

Another intriguing observation is that MST may require a greater number of treatment sessions to achieve remission compared to ECT. This finding hints at a differentiated “neuro-efficiency” profile, ECT may exploit therapeutic effect through high-intensity, broadly generalized neural perturbations per session (48), while MST might accomplish comparable clinical outcomes through a series of more focal, less intense, and cognitively sparing modulations (20). This paradigm aligns with the superior cognitive outcomes observed in our meta−analysis and suggests that MST’s clinical efficacy may be achieved through seizure activity with minimal electric field exposure, whereas ECT’s cognitive side effects may be partly attributable to its strong, widespread electric fields (14).

Several limitations of this review must be acknowledged. First, while blinding of treating personnel is inherently challenging due to the different physical procedures, participant blinding is feasible when treatments are delivered under general anesthesia. However, among the included studies, several did not implement adequate participant blinding, which introduces potential performance and detection bias, particularly for subjective outcomes (19, 30, 35). Second, there was considerable heterogeneity in technical parameters across studies, especially for ECT (e.g., electrode placement, pulse width), which likely contributes to the high heterogeneity in cognitive outcomes (I² = 94%). It is well established that different electrode placements (e.g., right unilateral vs. bifrontal vs. bitemporal), pulse widths (ultrabrief vs. brief), and dosing strategies differentially affect cognitive function. Third, the available literature lacks individual-patient data on seizure duration and its correlation with clinical improvement within the MST group, precluding a formal dose-response analysis. Fourth, our meta-analysis combined data from both randomized and non-randomized studies. The inclusion of non-randomized designs introduces a higher risk of selection bias and confounding, which may bias the pooled estimates. Although we assessed and reported risk of bias for each study, the methodological heterogeneity between study designs remains a limitation that warrants caution when interpreting the findings. Fifth, we pooled cognitive outcomes across different instruments (e.g., MoCA, RBANS) due to insufficient domain−specific data. Although SMD with a random−effects model is justifiable, the high heterogeneity (I² = 94%) limits interpretability. This finding is exploratory. Future studies should report domain−specific outcomes. Finally, long-term follow-up data beyond the acute treatment phase are limited, constraining conclusions about the durability of cognitive benefits and relapse prevention.

The collective evidence positions MST as a viable and often preferable advanced somatic therapy for MDD, particularly for patients in whom cognitive side effects are a paramount concern. Since the completion of our literature search (November 2025), a large, multicenter, double−blind, non−inferiority randomized controlled trial has been published (22). This study randomized 239 patients with treatment−resistant depression to receive either MST (n = 119) or right unilateral ultrabrief pulse ECT (n = 120), the latter representing a cognitively sparing form of ECT. The primary finding was that MST was non−inferior to ECT in antidepressant efficacy, with a significantly lower burden of subjective cognitive side effects. These results are highly concordant with our findings: comparable response and remission rates, and superior cognitive safety for MST. Notably, even against an optimized ECT comparator, MST maintained its cognitive advantage. The consistency between our meta−analysis of earlier studies and this large confirmatory trial reinforces the robustness of the conclusion that MST is a valuable alternative to ECT for patients with MDD, particularly when cognitive side effects are a primary concern. Future efforts to identify biological markers (e.g., neuroimaging signatures or quantitative electroencephalographic features) that differentiate response to MST versus ECT may enable more individualized treatment selection (49). Moreover, longer−term follow−up from controlled trials will be essential to determine whether the cognitive advantages of MST are sustained over time and whether these benefits translate into durable antidepressant effects over extended periods.

## 5 Conclusions

In conclusion, MST and ECT showed comparable response and remission rates in the treatment of MDD. Sensitivity analysis of depression scale score changes suggested potential superiority of ECT, though this result warrants cautious interpretation. MST consistently provided superior cognitive preservation, faster reorientation, and better overall tolerability, with fewer adverse events. These advantages make MST a valuable therapeutic alternative for patients with MDD, particularly when cognitive side effects are a primary concern. The shorter seizure duration with MST reflects its focal mechanism rather than a limitation. Further high-quality research is needed to optimize MST protocols and confirm long-term outcomes.

## Data Availability

The original contributions presented in the study are included in the article/**Supplementary Material**, further inquiries can be directed to the corresponding author/s.
