# Supplementary figures and images for: Comparative efficacy and cognitive safety of magnetic seizure therapy and electroconvulsive therapy in major depressive disorder: a systematic review and meta-analysis
Source: Front Psychiatry. 2026 Jun 17;17:1873016. doi: 10.3389/fpsyt.2026.1873016 (PMC13319006; doi:10.3389/fpsyt.2026.1873016)

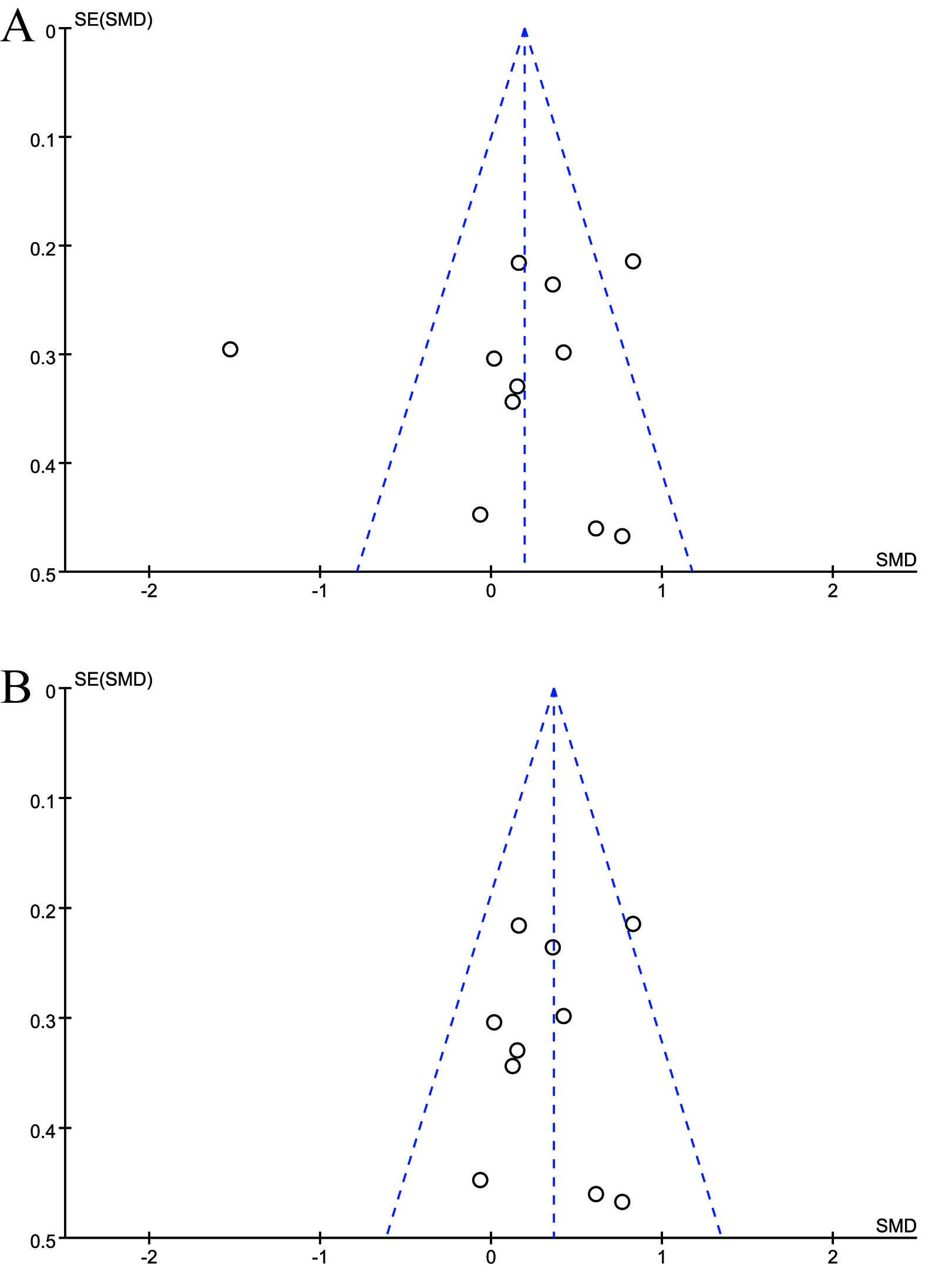

Supplement: Supplementary file 1 [file Image1.tif]
