# Supplementary material for: Comparative efficacy and cognitive safety of magnetic seizure therapy and electroconvulsive therapy in major depressive disorder: a systematic review and meta-analysis
Source: Front Psychiatry. 2026 Jun 17;17:1873016. doi: 10.3389/fpsyt.2026.1873016 (PMC13319006; doi:10.3389/fpsyt.2026.1873016)
Supplement: Supplementary file 3 [file Table2.docx]

| Title | The reasons for excluding |
| --- | --- |
| Magnetic Seizure Therapy In Bipolar Depression (MST-BpD)(1) | Full text unavailable |
| Cardiovascular Effects of High-Frequency Magnetic Seizure Therapy Compared With Electroconvulsive Therapy(2) | The primary outcome of the study was cardiovascular effects (such as changes in heart rate and blood pressure), and no antidepressant efficacy measures (such as HAMD, MADRS, etc.) or cognitive safety measures (such as MoCA, RBANS, etc.) were reported. Thus, they were not the focus of this review on efficacy or cognitive safety outcomes. |
| Fluctuations in resting motor threshold during electroconvulsive and magnetic seizure therapy(3) | This study focused on the fluctuation of resting motor threshold and was classified as a neurophysiological/therapeutic parameter study. It did not report any antidepressant efficacy or cognitive function outcome and did not meet the outcome inclusion criteria of this study. |
| Safety and feasibility of magnetic seizure therapy (MST) in major depression: randomized within-subject comparison with electroconvulsive therapy(4) | Although the study addressed efficacy and cognitive function of MST versus ECT, its design was an within-subject crossover comparison rather than a parallel-controlled design, as required for this review. In addition, the study did not provide independent between-group comparison data (such as independent means and standard deviations before and after treatment for each treatment group) that could be used for pooling in standard meta-analysis, and the sample size was very small (n=10), which was an early feasibility study and did not meet the quantitative pooling criteria of this study. |
| Comparison of electric field strength and spatial distribution of electroconvulsive therapy and magnetic seizure therapy in a realistic human head model(5) | This study was a computer-simulation model study (finite element simulation), was not a clinical trial, did not have samples of real patients, and did not report any clinical efficacy or cognitive outcomes; therefore, it was not eligible for inclusion in this review. |
| Comparable seizure characteristics in magnetic seizure therapy and electroconvulsive therapy for major depression(6) | The study design with within-subject crossover, no independent parallel control, the primary outcome being seizure characteristics rather than efficacy/cognitive indicators, the sample size was very small, and the population was non-responders of MST and there was selection bias, which did not meet the inclusion criteria of this systematic review and were excluded. |
| Cognitive Preservation and Antidepressant Efficacy of Magnetic Seizure Therapy in Adolescent Treatment Resistant Major Depressive Disorder in China: A Randomized Controlled Trial(7) | It was the same experiment as study Wang2025b(8), and the data were repeated. |
| Comparing the Neurocognitive Effects of Right Unilateral Ultra-Brief Pulse Electroconvulsive Therapy and Magnetic Seizure Therapy for the Treatment of Major Depressive Episode(9) | It was the same experiment as study Deng2024(10), and the data were repeated. |

1. Magnetic Seizure Therapy In Bipolar Depression (MST-BpD). Case Medical Research. 2019.

2. Zhang J-Y, Wu H, Jia L-N, Jiang W, Luo J, Liu Y, et al. Cardiovascular Effects of High-Frequency Magnetic Seizure Therapy Compared With Electroconvulsive Therapy. Journal of Ect. 2022;38(3):185-91.

3. Liu C, Liu S, Hu X, Guo Z, Xu Y. Fluctuations in resting motor threshold during electroconvulsive and magnetic seizure therapy. International Journal of Neuroscience. 2024.

4. Lisanby SH, Luber B, Schlaepfer TE, Sackeim HA. Safety and feasibility of magnetic seizure therapy (MST) in major depression: randomized within-subject comparison with electroconvulsive therapy. Neuropsychopharmacology : official publication of the American College of Neuropsychopharmacology. 2003;28(10):1852-65.

5. Lee WH, Lisanby SH, Laine AF, Peterchev AV. Comparison of electric field strength and spatial distribution of electroconvulsive therapy and magnetic seizure therapy in a realistic human head model. European Psychiatry. 2016;36:55-64.

6. Kayser S, Bewernick BH, Hurlemann R, Soehle M, Schlaepfer TE. Comparable seizure characteristics in magnetic seizure therapy and electroconvulsive therapy for major depression. European Neuropsychopharmacology. 2013;23(11):1541-50.

7. Wang W, Lu Y, Mi G-l, Qi S-f. Cognitive Preservation and Antidepressant Efficacy of Magnetic Seizure Therapy in Adolescent Treatment Resistant Major Depressive Disorder in China: A Randomized Controlled Trial. International Journal of Psychiatry in Medicine. 2025.

8. Wang W, Lu Y, Mi GL, Li XJ, Zhang DN, Qi SF. Cognitive preservation advantage and efficacy balance of magnetic seizure therapy in adolescent Major Depressive Disorder: a randomized controlled trial revealing efficacy cognition decoupling phenomenon. Riv Psichiatr. 2025;60(5):196-201.

9. McClintock SM, Deng Z-D, Husain MM, Thakkar VJ, Bernhardt E, Weiner RD, et al. Comparing the Neurocognitive Effects of Right Unilateral Ultra-Brief Pulse Electroconvulsive Therapy and Magnetic Seizure Therapy for the Treatment of Major Depressive Episode. Biological Psychiatry-Cognitive Neuroscience and Neuroimaging. 2025;10(2):175-85.

10. Deng ZD, Luber B, McClintock SM, Weiner RD, Husain MM, Lisanby SH. Clinical Outcomes of Magnetic Seizure Therapy vs Electroconvulsive Therapy for Major Depressive Episode: A Randomized Clinical Trial. JAMA Psychiatry. 2024;81(3):240-9.
